# Second-look arthroscopic and magnetic resonance analysis after internal fixation of osteochondral lesions of the talus

**DOI:** 10.1038/s41598-022-14990-5

**Published:** 2022-06-27

**Authors:** Young Rak Choi, Bom Soo Kim, Yu Mi Kim, Jae Yong Park, Jae Ho Cho, Joong Taek Ahn, Hyong Nyun Kim

**Affiliations:** 1 Department of Orthopedic Surgery, Asan Medical Center, University of Ulsan College of Medicine, Seoul, Republic of Korea; 2 Department of Orthopedic Surgery, Inha University Hospital, Incheon, Republic of Korea; 3 Department of Orthopedic Surgery, Sanbon Hospital, Wonkwang University College of Medicine, Gunpo-si, Gyeonggi-do Republic of Korea; 4 Department of Orthopedic Surgery, Hallym Sacred Heart Hospital, Hallym University College of Medicine, Anyang-si, Gyeonggi-do Republic of Korea; 5 Department of Orthopedic Surgery, Chuncheon Sacred Heart Hospital, Hallym University College of Medicine, Chuncheon-si, Gangwon-do Republic of Korea; 6 Department of Orthopedic Surgery, Kangnam Sacred Heart Hospital, Hallym University College of Medicine, 1, Shingil-ro, Yeongdeungpo-gu, Seoul, 07441 Republic of Korea

**Keywords:** Outcomes research, Diseases

## Abstract

The purpose of this study was to evaluate cartilage quality after internal fixation of osteochondral lesion of the talus (OLT) using second-look arthroscopies and MRIs. Thirty-four patients underwent internal fixation of OLTs involving large bone fragments. Twenty-one of these patients underwent second-look arthroscopies and 23 patients underwent MRIs postoperatively. The arthroscopic findings were assessed using the International Cartilage Repair Society (ICRS) grading system, and the MRI findings were evaluated using the Magnetic Resonance Observation of Cartilage Repair Tissue (MOCART) score. Five of the patients who underwent second-look arthroscopies showed normal cartilage, 12 showed nearly normal cartilage, 3 showed abnormal cartilage, and 1 showed severely abnormal cartilage, according to the overall ICRS repair grades. All the patients who achieved bone fragment union showed normal, or nearly normal cartilage upon second-look arthroscopy. The ICRS and MOCART scores were significantly higher for the patients with bone fragment union compared to those with nonunion (ICRS scores: 10.3 ± 1.5 vs. 6.0 ± 2.0, *p* < 0.001, MOCART score: 88.3 ± 10.0 vs. 39.0 ± 20.4, *p* < 0.001). Low signal intensities of the bone fragments on preoperative T1-weighted MRIs were not associated with nonunion (Fisher's exact test, *p* = 0.55), and the signal intensities increased postoperatively to levels similar to the underlying talus when bone union was achieved. Second-look arthroscopy and MRI showed normal, or nearly normal, cartilage after internal fixation of OLTs when bone union was achieved. The nonunion of bone fragments resulted in inferior cartilage quality.

## Introduction

An arthroscopic microfracture is the most frequently performed procedure for a symptomatic osteochondral lesion of the talus (OLT)^1–4^. Although the short- to mid-term clinical outcomes are generally good, the quality of the regenerated cartilage is unpredictable. There are also certain limitations to the procedure, in that it cannot restore the native hyaline cartilage and, for a large-sized lesion, the congruency of the talar dome cannot be restored^1–3,5,6^. More-invasive procedures, such as osteochondral autografting or allografting, can replace the lesion with the hyaline cartilage^7–10^, however, these replacement procedures are not without risks or complications^7,11^.

In the case of OLT involving large bone fragments, internal fixation of the osteochondral fragments may, in theory, be the best option, on the premise that bone union can be achieved, because the procedure can restore the natural congruency of the joint surface with the innate hyaline cartilage and maintain the subchondral bone without any donor site morbidity^12–16^. However, there are limited reports on the clinical outcomes of the procedure because OLTs with bone fragments large enough for healing are rarely encountered^16–21^. In a recent study on 25 patients with OLTs, bone peg fixation showed satisfactory clinical and radiographic results^22^. In a similar study on 26 patients who underwent internal fixation of OLTs, 77% achieved bone union with significant improvement of clinical outcomes^17^. However, the quality of the cartilage after bone fragment healing has not been assessed in these studies^17,22^. To the best of our knowledge, only one study has reported on the second-look arthroscopic findings of 15 ankles after internal fixation of OLTs^20^. This study showed stable and near-normal cartilage in all the ankles, but the authors could not confirm bone-to-bone healing because CT scans could not be obtained for the patients.

We evaluated cartilage quality after internal fixation of OLTs using second-look arthroscopies and MRIs. We also compared the quality of the cartilage between the patients that achieved bone fragment union and those that did not achieve union. Furthermore, as surgeons are concerned about whether relatively small chronic bone fragments inside the joint can heal on the talus after internal fixation, we compared the viability of bone fragments shown on preoperative T1-weighted MRIs with those seen on postoperative MRIs^23–25^. We hypothesized that the quality of the cartilage will be satisfactory after internal fixation of OLTs when bone fragment union is achieved.

## Materials and methods

### Patients

Between August 2014 and December 2019, a total of 34 patients (34 cases) underwent internal fixation of OLTs involving a large bone fragment. Among them, 21 patients agreed to undergo a second-look arthroscopy, and 23 agreed to have a postoperative MRI evaluation. Our institutional review board approved this study, and the patients provided informed consent. The study was conducted according to the relevant guidelines and regulations. The indication for internal fixation was a large OLT with a bone fragment at least 10 mm in diameter and 3 mm in depth on a computed tomography (CT) scan that failed radiologic union after 1 month of non-weight-bearing in a cast and 3 months of conservative treatment^26^. Contraindications included fragmentation of the bone fragment on a CT scan and patients with osteoarthritis or infectious pathologies. Preoperatively, CT scans were obtained to assess the size, location, shape, and morphology of the lesions. The thickness was measured as the depth of the lesion in the coronal CT images. The area was calculated using the ellipse formula of coronal length × sagittal length × 0.79^16^. MRIs were used to assess the condition of the bone fragments and cartilage. The conditions of the bone fragments were determined with preoperative T1-weighted MRIs, either as iso-signal or low signal intensities, compared to the underlying talus. A low signal intensity finding was interpreted as a non-viable fragment but was not considered to be a contraindication for internal fixation^25^. Acute osteochondral fractures were not included in the study as they were considered as different entities.

### Operative techniques

**Figure 1:**
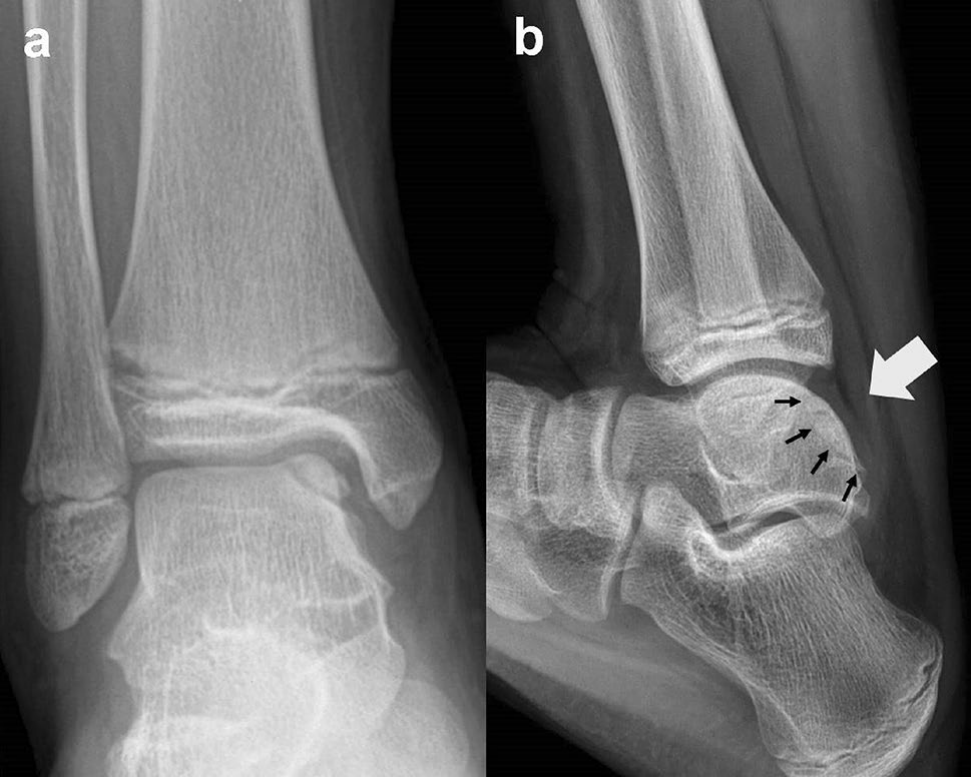
(**a**) A 13-year-old male patient with a large osteochondral fragment located on the medial talar dome was treated by internal fixation. **(b**) Maximal dorsiflexion of the ankle exposed the osteochondral fragment out of the tibiotalar articulation, which enabled internal fixation through a posteromedial arthrotomy without a malleolar osteotomy. The black arrows indicate the margin of the lesion. The white arrow indicates that the lesion could be approached from the posterior side through an arthrotomy.

The patients were placed in a supine position after spinal or general anesthesia was administered. A standard ankle arthroscopic examination was performed to assess the condition of the cartilage overlaying the lesion. A 3 cm longitudinal incision was made on the posterior border of the medial malleolus between the posterior tibial and flexor digitorum longus tendons. Maximal dorsiflexion of the ankle exposed the osteochondral fragment out of the tibiotalar articulation for better exposure (Fig. 1).

**Figure 2:**
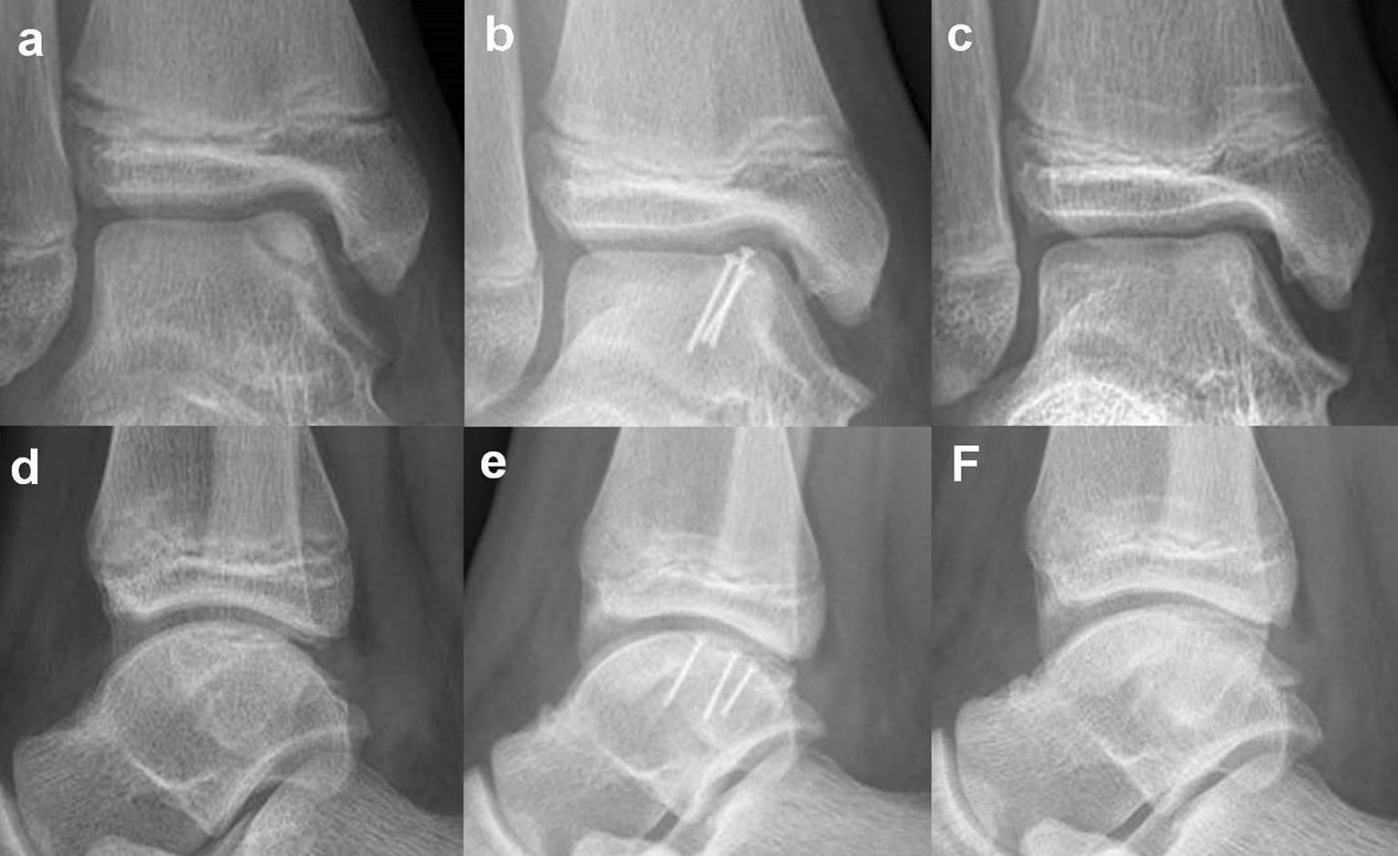
(**a**), (**d**) A large osteochondral fragment was located on the medial talar dome. (**b**), (**e**) Metal screws were inserted for firm fixation. (**c**), (**f**) When bone union was confirmed, the metal screws were removed.

The osteochondral fragment was detached from the talus and taken out of the ankle. Sometimes, anteromedial arthrotomy was required to detach the anterior part of the fragment. The crater on the talus was examined, and fibrotic tissue and sclerotic bone were removed with a curette and burr. The base of the fragment was examined, and the fibrous tissue was removed, taking care not to break the fragment. Cancellous bone was harvested from the lateral calcaneus and impacted on the base of the defect. The osteochondral fragment was reduced on the crater, and a 3.0 mm Bio-Compression Screw (Arthrex Inc., Naples, USA) was fixed at the center of the lesion. When the fragment was relatively thin (depth < 4.0 mm), 1.5 mm metal screws were used as a precaution, as 3.0 mm bioabsorbable screws may have broken the fragment (Fig. 2).

A below-knee cast was applied for 6 weeks postoperatively. Tolerable weight-bearing began 8 weeks postoperatively.

### Radiological evaluation of bone union

At 6 months postoperatively, a CT scan was checked to assess for bone fragment healing. When the bone fragment reached > 75% continuity with the talus on the sagittal, coronal, and axial images showing the largest bone fragment, bone union was considered to have been achieved^27^.

### Second-look arthroscopic surgery

**Figure 3:**
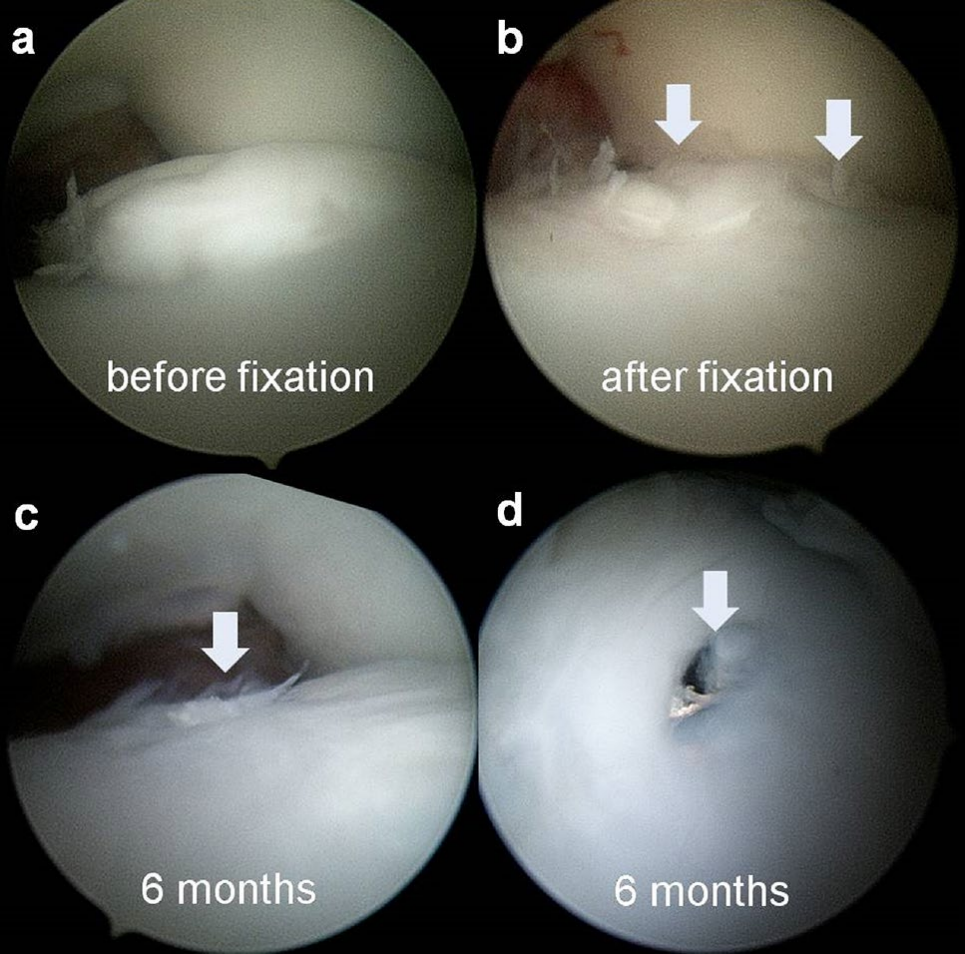
(**a**) A diagnostic arthroscopy shows a large-sized osteochondral fragment protruding from the medial talar dome. (**b**) The osteochondral fragment was reduced and stabilized with two metal screws. (**c**) A second-look arthroscopic procedure was performed 6 months after the internal fixation, during the metal screw removal surgery. The osteochondral fragment was congruent with the surrounding cartilage. Smooth hyaline-like cartilage was observed. (**d**) The metal screws were located for removal. The arrows show where the metal screws were inserted.

When bone union was confirmed on a CT scan, the metal screws were removed and, at the time of the screw removal, a second-look arthroscopy was performed with ankle joint traction to assess the quality of the cartilage according to the International Cartilage Repair Society (ICRS) grading system and the Oswestry Arthroscopy Score (OAS) (Fig. 3)^28,29^. The ICRS score and OAS are both reliable and relevant scores validated for macroscopic evaluation of cartilage repair^28,29^. When revision surgery was required for cases with bone fragment nonunion, a second-look arthroscopy was performed at the time of the surgery. The ICRS scores and OASs were compared between the patients that achieved bone union and those that did not. The clinical results were assessed using the Foot Function Index (FFI)^30^ at the time of the second-look arthroscopic surgery to evaluate the correlation between the clinical outcomes and the ICRS scores and OASs. The FFI is a validated, patient-assessed questionnaire containing the following three subscales: pain, disability, and activity limitations (total possible score of 100 points, with 100 being the worst)^30^.

### MRI assessment

Postoperative MRIs were performed with a fat-saturated, 3-dimensional, fast-spoiled gradient recalled acquisition in the steady state (repetition time/echo time (TR/TE) 40 ms/6 ms; 1.5 mm slice thickness; 150 mm FOV; 0.75 excitations) and T2-weighted turbo spin-echo sequence to analyze the articular cartilage^6^. T1-weighted images were also obtained with a TR/TE of 600-700/9-12, 2 mm slice thickness and 150 mm FOV to evaluate the conditions of the bone fragments. The Magnetic Resonance Observation of Cartilage Repair Tissue (MOCART) scoring system was used to evaluate the recovery of the cartilage and bone fragments^31,32^. The MOCART is a validated assessment tool used to perform structured morphological assessments of articular cartilage repairs, with 100 set as the best and 0 the worst possible score^31,32^. The MRI findings were also evaluated using criteria developed for the assessment of osteochondral autograft stransplants^7^. The conditions of the bone fragments were determined using T1-weighted MRIs and defined as either iso-intensity or low intensity, compared to the underlying talus. The MRI findings were compared between the patients that achieved bone union and those that did not. The clinical outcomes were assessed at the time of the MRIs using the FFIs to evaluate the correlation between the MOCART scores and the clinical outcomes.

### Statistical analysis

All the data are expressed as the mean ± standard deviation. The ICRS and MOCART scores and the OAS were compared between the patients that achieved bone union and those that did not using the independent t-test and Mann–Whitney U test. The relationship between the ICRS and the OAS, MOCART, and clinical outcome scores (FFI) was examined using Pearson's correlation coefficient. The association between the low signal intensities of the bone fragments on the preoperative T1-weighted MRIs and the incidence of bone fragment nonunion was examined using the Fisher’s exact test. SPSS version 21.0 (IBM Corporation, Armonk, NY, USA) was used for the statistical analyses. Differences with *p* values < 0.05 were considered statistically significant.

### Ethics approval

This study was approved by Institutional Review Board (IRB) of Hallym University Kangnam Sacred Heart Hospital (IRB- 2020-02-027).

## Results

Thirty-four patients (20 male, 14 female), with a median age of 16 years (range, 11 to 29 years) underwent internal fixation of OLTs involving a large bone fragment. Twenty-two lesions were located in the centromedial talus, and 12 lesions were located in the posteromedial talus. The mean size of the fragment measured on the CT scan was 12.4 ± 1.7 mm in sagittal length, 8.2 ± 1.8 mm in coronal length, 4.4 ± 0.9 mm in depth, and 79.8 ± 21.4 mm^2^ in area. A total of 28 (82%) patients achieved radiologic bone union on postoperative CT scan. Metal screws were used in 17/34 (50%) cases. The results are summarized in Supplementary Table 1. Twenty-one patients underwent second-look arthroscopy at a mean of 8.8 months (range, 6 to 45 months) postoperatively (Fig. 3). A total of 15 s-look arthroscopies were combined with metal-screw removal after bone fragment union, 5 were combined with revision surgery because of bone fragment nonunion, and 1 was combined with lateral ligament repair. During the second-look arthroscopy for these patients, 5 showed normal cartilage, 12 showed nearly normal cartilage, 3 showed abnormal cartilage, and 1 showed severely abnormal cartilage, according to the overall ICRS repair grades. Among the 16 patients that achieved bone union, 5 showed normal cartilage and 11 showed nearly normal cartilage, whereas, among the 5 patients with nonunion, 1 showed nearly normal cartilage, 3 showed abnormal cartilage, and 1 showed severely abnormal cartilage (Table 1).

**Table 1:** Comparison of the second-look arthroscopic findings between the patients with bone union and nonunion after internal fixation.

	Bone union group (*n* = 16)^a^	Non-union group (*n* = 5)^a^	*p*-value
Age (yrs)	15.1 ± 3.6	17.6 ± 3.8	0.20
Sex (male/female)	8/8	4/1	0.34
Fragment size on CT scan (mm^2^)	74.0 ± 18.8	65.5 ± 9.5	0.35
**ICRS grade**^**b**^			
Grade I (normal)	5 (23.8%)	0 (0%)	0.001
Grade II (nearly normal)	11 (52.4%)	1 (4.8%)	
Grade III (abnormal)	0 (0%)	3 (14.3%)	
Grade IV (severely abnormal)	0 (0%)	1 (4.8%)	
ICRS score (0–12)	10.3 ± 1.5	6.0 ± 2.0	< 0.001
OAS (0–10)	8.8 ± 0.9	5.2 ± 2.3	0.02
FFI (0–100)	12.7 ± 6.4	27.0 ± 6.6	< 0.001

^a^Values are given as the mean ± standard deviation, with the exception of sex and ICRS grade. ^b^Values are given as the number and the percentage in parenthesis.

*ICRS* International Cartilage Repair Society (total score of 12 points, with 12 being the best), *OAS* Oswestry Arthroscopy Score (total score of 10 points, with 10 being the best), *FFI* Foot Function Index (total score of 100 points, with 100 being the worst).

**Figure 4:**
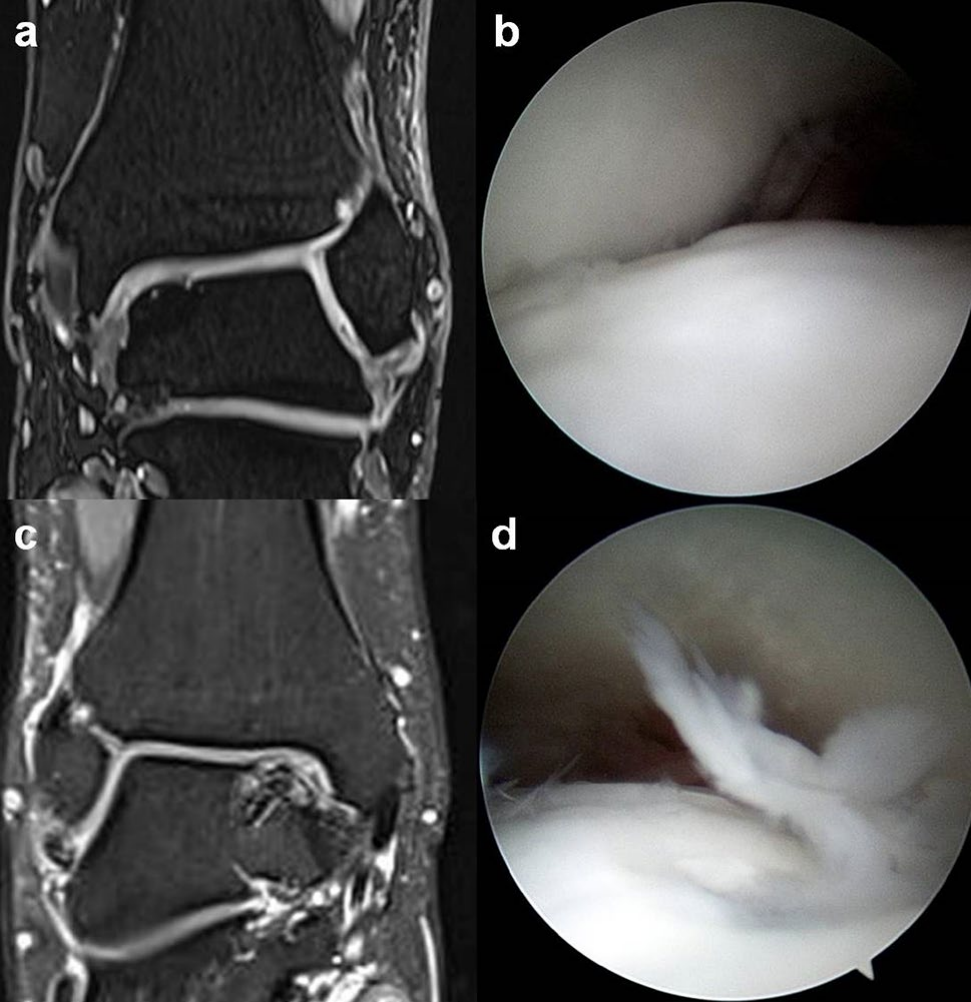
(**a**) The postoperative MRI of an 18-year-old female patient with bone fragment union shows normal congruity of the fixed osteochondral fragment without any cysts underneath the fragment. (**b**) The second-look arthroscopic image shows smooth, pearly, hyaline-like cartilage of the osteochondral fragment, which is well integrated with surrounding cartilage. (**c**) The postoperative MRI of a 21-year-old male patient with bone fragment nonunion shows incongruity of the fragment with irregular cartilage and cyst underneath the fragment. (**d**) The second-look arthroscopic image shows severe fibrillation and flap tearing of the cartilage.

The ICRS scores and OASs were significantly higher for the patients with bone union compared to those with nonunion (ICRS scores: 10.3 ± 1.5 vs. 6.0 ± 2.0, *p* < 0.001, OAS: 8.8 ± 0.9 vs. 5.2 ± 2.3, p = 0.02) (Table 1), (Fig. 4), (Supplementary Tables 1, 2, 3). The FFIs were negatively correlated to the ICRS scores (Pearson’s correlation coefficient, r =  − 0.65, *p* = 0.001) and OASs (Pearson’s correlation coefficient, r =  − 0.66, *p* = 0.001). The difference in the ICRS scores and OASs between the patient with skeletally immature ankle and those with skeletally mature ankle was not statistically significant (Table 2).

**Table 2:** Comparison of secondary arthroscopic findings between patients with skeletally immature and mature ankle.

	Skeletally immature (*n* = 10)^a^	Skeletally mature (*n* = 11)^a^	*p*-value
Age (yrs)	12.7 ± 0.9	18.5 ± 3.0	< 0.001
Sex (male/female)	5/5	7/4	0.97
Lesion size (mm^2^)	69.7 ± 19.4	74.1 ± 15.7	0.57
Bone union	8 (80%)	8 (73%)	1.00
**ICRS grade**^**b**^			
Grade I (normal)	4 (19%)	1 (5%)	0.12
Grade II (nearly normal)	5 (24%)	7 (33%)	
Grade III (abnormal)	0 (0%)	3 (14%)	
Grade IV (severely abnormal)	1 (5%)	0 (0%)	
ICRS score (0–12)	10.0 ± 2.8	8.6 ± 2.0	0.21
OAS (0–10)	8.4 ± 2.4	7.5 ± 1.6	0.30
FFI (0–100)	16.0 ± 9.4	16.1 ± 8.7	0.98

Skeletal maturity was determined by whether the growth plate of the distal tibia was opened or closed.

^a^Values are given as the mean ± standard deviation, with the exception of sex, bone union and ICRS grade.

^b^Values are given as the number and the percentage in parenthesis.

*ICRS* International Cartilage Repair Society, *OAS* Oswestry Arthroscopy Score, *FFI* Foot Function Index.

Twenty-three patients underwent MRIs at a mean of 28.3 months (range, 5 to 56 months) postoperatively. The mean MOCART scores were significantly higher for the patients with bone union compared to those with nonunion (88.3 ± 10.0 vs. 39.0 ± 20.4, *p* < 0.001) (Supplementary Table 4). The difference in MOCART scores between the patient with skeletally immature ankle and those with skeletally mature ankle was not statistically significant (82.7 ± 23.0 vs. 71.0 ± 25.4, *p* = 0.26). The MRI evaluations, using the Imhoff criteria^13^, showed better results for the patients with bone union compared to those with nonunion (Table 3).

**Table 3:** The MRI evaluations according to the Imhoff criteria.

Criteria	Category	Subcategory	No. of patients^a^		*p* value
			Bone union group (*n* = 18)	Non-union group (*n* = 5)	
Osteochondral fragment	Congruency	Normal	16 (69.6%)	2 (8.7%)	0.02
		Minor incongruency	1 (4.3%)	0 (0%)	
		Major incongruency	1 (4.3%)	3 (13.0%)	
	Cartilage	Normal	10 (43.5%)	0 (0%)	0.046
		Signal changes	8 (34.8%)	5 (21.7%)	
		Superficial defect	0 (0%)	0 (0%)	
		Substance defect	0 (0%)	0 (0%)	
	Subchondral bone	Normal	17 (73.9%)	0 (0%)	< 0.001
		Edema	1 (4.3%)	3 (13.0%)	
		Cysts	0 (0%)	2 (8.7%)	
		Large defect	0 (0%)	0 (0%)	
Surroundings	Cartilage	Normal	17 (73.9%)	1 (4.3%)	0.003
		Signal changes	1 (4.3%)	4 (17.4%)	
		Superficial defect	0 (0%)	0 (0%)	
		Substance defect	0 (0%)	0 (0%)	
	Subchondral bone	Normal	14 (60.9%)	0 (0%)	0.002
		Edema	4 (17.4%)	3 (13.0%)	
		Cysts	0 (0%)	2 (8.7%)	
		Large defect	0 (0%)	0 (0%)	
Tibia	Cartilage	Normal	17 (73.9%)	1 (4.3%)	0.001
		Signal changes	1 (4.3%)	3 (13.0%)	
		Superficial defect	0 (0%)	1 (4.3%)	
		Substance defect	0 (0%)	0 (0%)	
Effusion		None	14 (60.9%)	1 (4.3%)	0.02
		Mild	4 (17.4%)	3 (13.0%)	
		Large	0 (0%)	1 (4.3%)	

^a^Values are given as the number and the percentage in parenthesis.

**Figure 5:**
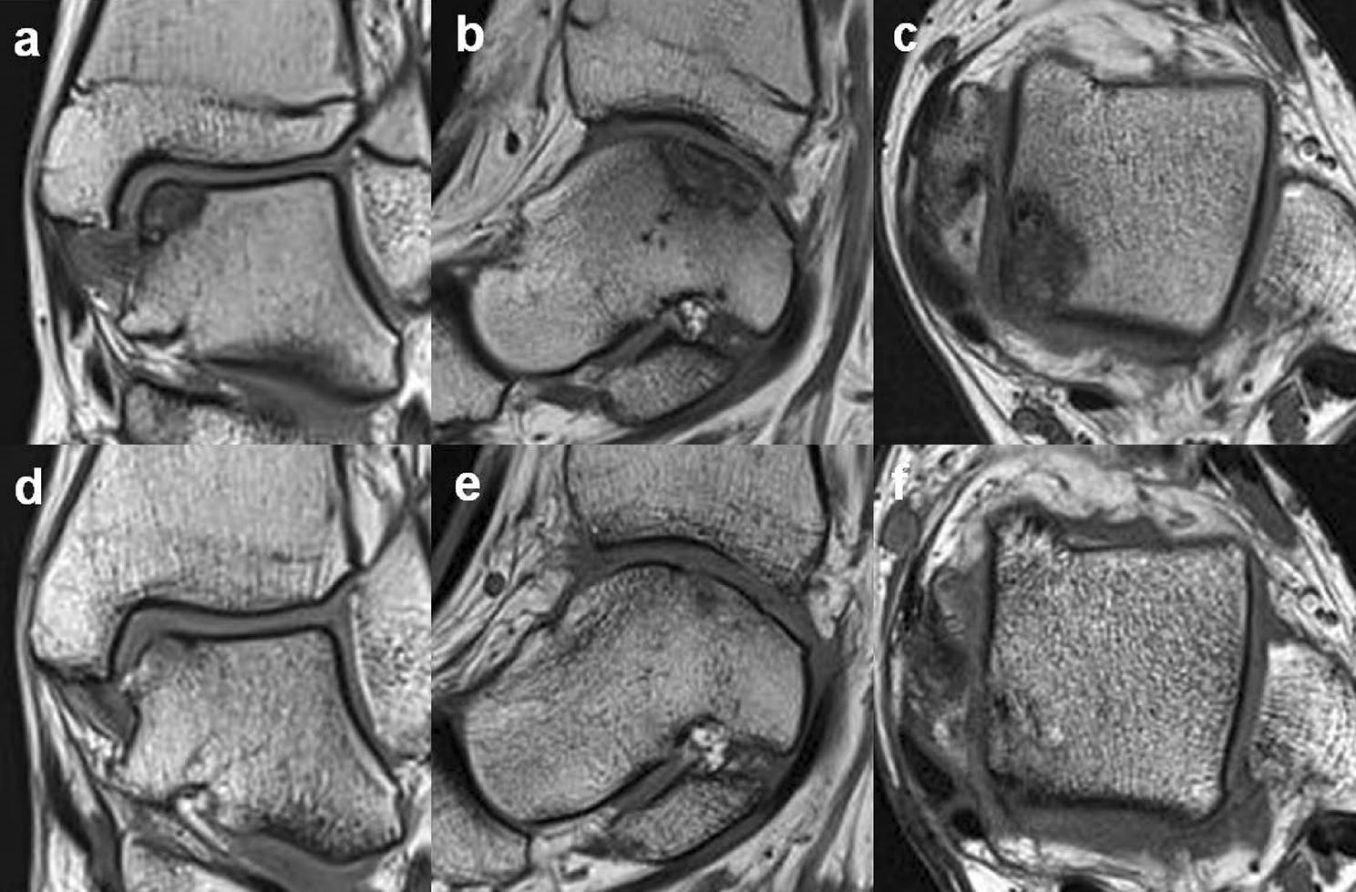
Preoperative T1-weighted MRI scans of a 15-year-old male patient show low signal intensities of the osteochondral fragment on (**a**) coronal, (**b**) sagittal, and **(c**) axial images. The fragment appeared non-viable for bone healing. (**d**), (**e**), (**f**) On the 24-month postoperative MRIs, the signal intensities increased to levels similar to the underlying talus.

The FFIs were negatively correlated to the MOCART scores (Pearson’s correlation coefficient, r =  − 0.75, *p* < 0.001). The preoperative low signal intensities of the T1-weighted MRIs of the bone fragments that appeared non-viable for healing were not associated with nonunion (Fisher's exact test, *p* = 0.55). When bone union was achieved and confirmed on 6 months CT scans, all the patients with low signal intensities on the preoperative T1-weighted MRIs showed postoperative MRI signal increases to levels similar to the underlying talus (Fig. 5). The bone fragment signal intensities on the preoperative T1-weighted MRIs were not associated with the postoperative T1-weighted MRI (Fisher's exact test, *p* = 0.55). None of the patients showed neurologic symptoms or other complications.

## Discussion

The most important finding of the current study is that the second-look arthroscopies and postoperative MRIs showed normal, or nearly normal cartilage after internal fixation of the OLTs when bone union was achieved. However, bone fragment nonunion resulted in inferior cartilage quality. The bone fragments with low signal intensities on the preoperative T1-weighted MRIs that seemed non-viable were not associated with nonunion (Fisher's exact test, *p* = 0.55), and the signal intensities seen on the postoperative MRIs of these fragments increased to levels similar to those of the underlying talus after bone union was achieved.

Second-look arthroscopy is effective for evaluating the recovery of articular cartilage in patients with OLTs^5,6^. It has advantages over postoperative MRIs for assessing cartilage quality, in that the firmness of the cartilage can be assessed using a probe, and direct observation of the color and smoothness of the cartilage is possible^33,34^. However, there are limited reports on second-look arthroscopic findings after surgical procedures for OLT^5,6,9^. In a study of second-look arthroscopies on 25 patients after microfracture of OLTs, 36% of the lesions were incompletely healed and showed inferior repair tissue quality after a mean postoperative follow-up of 3.6 years^6^. To overcome the limitations of microfractures that are unable to restore native hyaline cartilage, a more invasive procedure, such as osteochondral autografting, is suggested for larger lesions^7,9,10^. Second-look arthroscopy on 16 ankles after osteochondral autografting revealed osteochondral graft consistency and congruity between the graft and the native cartilage in 14 (87.5%) ankles a mean of 3.4 years postoperatively^9^. In the present study, the second-look arthroscopy was performed in 21 ankles after internal fixation of OLTs that showed normal, or nearly normal cartilage when bone union was achieved.

Although second-look arthroscopy is an excellent procedure for the morphological evaluation of cartilage, it does not allow for evaluation of the bone underneath the lesion^5,33^. We obtained MRI scans a mean of 28.3 months postoperatively to evaluate the patients’ cartilage quality, as well as the status of the bone underneath the cartilage. The mean MOCART score was significantly higher for patients with bone union compared to those with nonunion (88.3 ± 10.0 vs. 39.0 ± 20.4, *p* < 0.001) (Supplementary file 4). Imhoff et al.^7^ developed MRI criteria for assessing outcomes after osteochondral autografting^7^. As the criteria were developed to evaluate transplanted osteochondral plugs involving the cartilage, subchondral bone, and the structures surrounding the transplant, it would be appropriate to use these criteria to evaluate osteochondral fragments after internal fixation. In an MRI study^7^ using the criteria to evaluate outcomes after osteochondral autografting in 26 cases of OLT, 23.1% showed normal congruity of the transplanted osteochondral plug, whereas, in our study, 78.3% showed normal congruity of the fixed osteochondral fragment (Table 3). In cases of osteochondral autografting, 46.2% showed cysts underneath the transplanted osteochondral plugs, and 19.2% showed large defects^7^, whereas 8.7% of the patients in the present study showed cysts underneath the osteochondral fragments, with no large defects after internal fixation (Table 3).

For a small chronic bone fragment inside the joint, such as a non-united scaphoid bone fracture fragment, viability of the fragment can be a concern for achieving bone union^23–25^. The bone fragment completely detached from the talus can result in avascular necrosis^35,36^. The possibility of further fragmentation and degeneration of this bone fragment over time could be the reason why the cases of fixation in eldery patients are rare. Although a bone fragment of at least 10 mm in diameter and 3 mm in depth on CT scans is relatively large compared to other commonly encountered OLTs, it is still considered a small bone fragment, and when there is an indication of decreased vascularity, surgeons are concerned about the possibility of nonunion^26^. The vascular status of a small bone fragment such as this is detectable by T1-weighted MRI^25^. In the present study, the bone fragments with low preoperative T1-weighted MRI signal intensities that seemed not to be viable were not associated with nonunion (Fisher's exact test, *p* = 0.55), and the postoperative MRI signal intensities increased to levels similar to the underlying talus once bone union was achieved. The signal intensities of the bone fragments on the preoperative T1-weighted MRIs were not associated with those of the postoperative T1-weighted MRIs (Fisher's exact test, *p* = 0.55). This may indicate that, although an osteochondral fragment may not seem viable for bone healing on a preoperative MRI, it is possible for it to heal after internal fixation, and a low signal intensity on a preoperative T1-weighted MRI should not be a contraindication for the procedure. However, it should be noted that the presence of a low signal intensity on a T1-weighted images could reflect either a non-viable bone or bone marrow edema pattern. In a direct comparison between the accuracy of unenhanced and contrast-enhanced MRI in evaluating the viability of non-united scaphoid bone fractured fragments, contrast-enhanced MRI showed significantly higher sensitivity, specificity and accuracy^37^. Recent studies have suggested that dynamic contrast-enhanced MR examinations can assess the viability of small bones with much better accuracy^38^. A future study with dynamic gadolinium-enhanced MRI may confirm our findings. Furthermore, as continued development in the field of quantitative MRI is enabling the non-invasive assessment of collagen orientation and proteoglycan content within the repaired cartilage; a future study involving quantitative MRI techniques like T1 and T2 relaxometry, diffusion weighted imaging, and magnetization transfer may further confirm our findings^39^.

This study was limited by the small number of subjects and short follow-up period. However, a large sized OLT indicated for fixation is rarely encountered. A small number of subjects is inevitable for studies such as this, where there is a low incidence of the disorder. In a study involving 1,068,215 people aged 2 to 19 years from the database of a large health care system, only 85 patients had ankle osteochondritis dissecans (OCD), while 206 patients had knee OCD^40^. Among the 85 patients with ankle OCD, 27 (32%) required surgery and only 4 (5%) underwent fixation. In the present study, the second-look arthroscopies were performed for only 61.8% of the patients who underwent internal fixation of OLTs, which may predispose this study for selection bias. However, a low participation rate for second-look arthroscopies is inevitable because patients without any symptoms, or those with only minor symptoms, are reluctant to undergo additional surgeries. In a recently published report on second-look arthroscopies performed after surgical treatment of OLTs, the participation rate was 18%^6^. Another limitation was that the findings between the second-look arthroscopy procedures and the postoperative MRIs could not be compared because they were obtained during different time periods. A future study with a larger population, longer follow-up period, and a histological analysis may further confirm the present findings.

## Conclusions

Second-look arthroscopies and MRIs showed normal, or nearly normal cartilage after internal fixation of OLTs when bone union was achieved. However, bone fragment nonunion resulted in inferior cartilage quality.

## Supplementary Information

Supplementary Information 1.

Supplementary Information 2.

Supplementary Information 3.

Supplementary Information 4.

## Data Availability

The datasets used and/or analysed during the current study are available from the corresponding author on reasonable request.
