# Supplementary material for: Second-look arthroscopic and magnetic resonance analysis after internal fixation of osteochondral lesions of the talus
Source: Sci Rep. 2022 Jun 27;12:10833. doi: 10.1038/s41598-022-14990-5 (PMC9237059; doi:10.1038/s41598-022-14990-5)
Supplement: Supplementary file 1 — Supplementary Information 1. [file 41598_2022_14990_MOESM1_ESM.docx]

**Supplementary Table 1**. Details and Results for the 27 Patients

| **Case** | **Sex** | **Age (yrs)** | **Growth plate^a^** | **Stage^b^** | **Location** | **Size of fragment (mm)^c^** | **Approach** | **Fixation type** | **Bone Union** | **ICRS scores/ grade^d^** | **OAS^e^** | **MOCART score^f^** |
| --- | --- | --- | --- | --- | --- | --- | --- | --- | --- | --- | --- | --- |
| **1** | M | 13 | Opened | III | PM | 13.2×6.2×4.1 | P | AP | No | 3 / IV | 2 | 15 |
| **2** | F | 20 | Closed | III | CM | 11.1×9.0×3.4 | P | AP | No | 5 / III | 4 | 30 |
| **3** | F | 13 | Opened | III | CM | 14.1×6.7×4.2 | P | AS | Yes | - | - | 95 |
| **4** | M | 21 | Closed | III | PM | 10.3×8.3×3.4 | P | MS | No | 7 / III | 6 | 45 |
| **5** | M | 14 | Opened | III | PM | 12.6×5.3×4.3 | P | AS | No | 8 / II | 8 | 70 |
| **6** | F | 11 | Opened | III | CM | 11.8×6.4×4.7 | P | AS | Yes | 12 / I | 10 | 90 |
| **7** | M | 20 | Closed | II | CM | 14.4×5.6×4.9 | P | AS | No | 7 / III | 6 | 35 |
| **8** | F | 15 | Closed | III | PM | 14.1×7.2×4.3 | P | MS | Yes | 11 / II | 8 | 90 |
| **9** | M | 13 | Opened | IV | PM | 15.7×9.6×4.9 | B | MS | Yes | 12 / I | 10 | 95 |
| **10** | F | 15 | Closed | III | PM | 13.7×6.5×3.8 | P | MS | Yes | 10 / II | 8 | - |
| **11** | M | 18 | Closed | IV | PM | 14.3×7.3×5.2 | P | AS | Yes | - | - | 75 |
| **12** | M | 16 | Closed | III | CM | 11.3×11.2×5.3 | B | MS | Yes | 9 / II | 8 | 85 |
| **13** | M | 14 | Opened | III | CM | 14.1×10.2×4.8 | P | AS | Yes | - | - | 75 |
| **14** | M | 18 | Closed | III | PM | 10.5×6.9×3.3 | P | MS | Yes | 8 / II | 8 | 70 |
| **15** | F | 15 | Closed | III | CM | 12.5×5.2×4.2 | B | MS | Yes | 10 / II | 9 | 100 |
| **16** | F | 29 | Closed | III | CM | 12.6×7.2×5.3 | P | AS | Yes | - | - | 90 |
| **17** | F | 12 | Opened | III | CM | 11.3×8.7×3.6 | P | MS | Yes | 11 / II | 9 | 95 |
| **18** | M | 18 | Closed | III | PM | 10.4×7.6×4.2 | P | MS | Yes | 8 / II | 8 | - |
| **19** | M | 15 | Closed | III | PM | 13.0×11.6×4.9 | P | AS | Yes | - | - | 90 |
| **20** | M | 24 | Closed | III | PM | 12.6×8.9×4.2 | P | MS | Yes | 8 / II | 7 | - |
| **21** | F | 12 | Opened | III | CM | 12.0×6.7×3.8 | P | MS | Yes | 12 / I | 10 | 100 |
| **22** | F | 13 | Opened | III | CM | 10.3×8.1×3.7 | P | MS | Yes | 10 / II | 9 | 95 |
| **23** | M | 12 | Opened | III | PM | 10.1×9.1×3.4 | P | MS | Yes | 10 / II | 8 | 75 |
| **24** | M | 14 | Opened | III | CM | 10.1×6.3×4.2 | B | MS | Yes | 10 / II | 9 | 95 |
| **25** | F | 13 | Opened | III | CM | 13.2×6.9×4.0 | P | MS | Yes | 12 / I | 9 | 100 |
| **26** | M | 15 | Opened | III | PM | 13.0×10.0×5.0 | P | AP | Yes | - | - | 75 |
| **27** | M | 21 | Closed | III | CM | 12.0×10.0×8.0 | MO | MS | Yes | 12 / I | 10 | - |

^a^ Growth plate of distal tibia; ^b^ Berndt and Harty Stage; ^c^ Size of a fragment is presented as sagittal length×coronal length×depth; ^d^ICRS, International Cartilage Repair Society scores at second-look arthroscopy are graded as Grade I: normal with the score of 12, Grade II: nearly normal with the score 11-8, Grade III: abnormal with the score of 7-4, Grade IV: severely abnormal with the score of 3-0; ^e^OAS, Oswestry Arthroscopy Score at second-look arthroscopy (total score of 10 points, with 10 being the best); ^f^MOCART, Magnetic Resonance Observation of Cartilage Repair Tissue (MOCART) scores (total score of 100 points, with 100 being the best)

M, male; F, female; PM, posteromedial; CM, centromedial; P, posterior; B, both from anterior and posterior; MO, malleolar osteotomy; AP, absorbable pin; AS, absorbable screw; MS, metal screw
