# Supplementary material for: Second-look arthroscopic and magnetic resonance analysis after internal fixation of osteochondral lesions of the talus
Source: Sci Rep. 2022 Jun 27;12:10833. doi: 10.1038/s41598-022-14990-5 (PMC9237059; doi:10.1038/s41598-022-14990-5)
Supplement: Supplementary file 2 — Supplementary Information 2. [file 41598_2022_14990_MOESM2_ESM.docx]

**Supplementary Table 2**. International Cartilage Repair Society (ICRS) score at second-look arthroscopy

| **Category** | **Points** | **No. of patients^a^** | | **p-value** |
| --- | --- | --- | --- | --- |
|  |  | **Bone union group (n=16)** | **Non-union group (n=5)** |  |
| **Degree of defect repair** |  |  |  | 0.24 |
| **In level with surrounding cartilage** | 4 | 16(76.2%) | 4 (19.0%) |  |
| **75% repair of defect depth** | 3 | 0 (0%) | 0 (0%) |  |
| **50% repair of defect depth** | 2 | 0 (0%) | 1 (4.8%) |  |
| **25% repair of defect depth** | 1 | 0 (0%) | 0 (0%) |  |
| **0% repair of defect depth** | 0 | 0 (0%) | 0 (0%) |  |
| **Integration to border zone** |  |  |  | 0.02 |
| **Complete integration with surround cartilage** | 4 | 5 (23.8%) | 0 (0%) |  |
| **Demarcating border <1mm** | 3 | 5 (23.8%) | 0 (0%) |  |
| **3/4th of graft integrated, 1/4th with a notable border >1mm width** | 2 | 6 (28.6%) | 3 (14.3%) |  |
| **1/2 of graft integrated with surrounding cartilage, 1/2 with a notable border >1mm** | 1 | 0 (0%) | 0 (0%) |  |
| **From no contact to 1/4th of graft integrated with surrounding cartilage** | 0 | 0 (0%) | 2 (9.5%) |  |
| **Macroscopic appearance** |  |  |  | 0.001 |
| **Intact smooth surface** | 4 | 6 (28.6%) | 0 (0%) |  |
| **Fibrillated surface** | 3 | 5 (23.8%) | 0 (0%) |  |
| **Small, scattered fissures or cracks** | 2 | 5 (23.8%) | 1 (4.8%) |  |
| **Several, small or few but large fissures** | 1 | 0 (0%) | 4 (19.0%) |  |
| **Total degeneration of grafted area** | 0 | 0 (0%) | 0 (0%) |  |
| **Overall repair assessment** |  |  |  | 0.001 |
| **Grade I: normal** | 12 | 5 (23.8%) | 0 (0%) |  |
| **Grade II: nearly normal** | 11-8 | 11 (52.4%) | 1 (4.8%) |  |
| **Grade III: abnormal** | 7-4 | 0 (0%) | 3 (14.3%) |  |
| **Grade IV: severely abnormal** | 3-0 | 0 (0%) | 1 (4.8%) |  |
| **Total scores^b^** |  | 10.3 ± 1.5 | 6.0 ± 2.0 | <0.001 |

^a^Values are given as the number of the patients with percentage in parenthesis. ^b^Total scores are given as the mean ± standard deviation. Bone union group: patients who achieve bone union of the osteochondral fragment, non-union group: patients who did not achieve bone union of the osteochondral fragment.
