# Supplementary material for: Second-look arthroscopic and magnetic resonance analysis after internal fixation of osteochondral lesions of the talus
Source: Sci Rep. 2022 Jun 27;12:10833. doi: 10.1038/s41598-022-14990-5 (PMC9237059; doi:10.1038/s41598-022-14990-5)
Supplement: Supplementary file 3 — Supplementary Information 3. [file 41598_2022_14990_MOESM3_ESM.docx]

**Supplementary Table 3**. Oswestry Arthroscopy Score (OAS) at second-look arthroscopy

| **Category** | **Points** | **No. of patients^a^** | | **p-value** |
| --- | --- | --- | --- | --- |
|  |  | **Bone union group (n=16)** | **Non-union group (n=5)** |  |
| **Graft level with surrounding cartilage** | | | | |
| **Level** | 2 | 14 (66.7%) | 2 (9.5%) | 0.054 |
| **Raised** | 1 | 2 (9.5%) | 2 (9.5%) |  |
| **Below** | 0 | 0 (0%) | 1 (4.8%) |  |
| **Integration with surrounding cartilage** | | | | |
| **Complete** | 2 | 10 (47.6%) | 0 (0%) | 0.01 |
| **Minor disruption (<25% of area)** | 1 | 6 (28.6%) | 3 (14.3%) |  |
| **Major disruption (>25% of area)** | 0 | 0 (0%) | 2 (9.5%) |  |
| **Appearance of surface** | | | | |
| **Smooth** | 2 | 5 (23.8%) | 0 (0%) | <0.001 |
| **Fine fronds** | 1 | 11 (52.4%) | 1 (4.8%) |  |
| **Severe fronds/fibrillation** | 0 | 0 (0%) | 4 (19.0%) |  |
| **Color of graft** | | | | |
| **Pearly, hyaline-like** | 2 | 16 (76.2%) | 2 (9.5%) | 0.048 |
| **White** | 1 | 0 (0%) | 3 (14.3%) |  |
| **Yellow bone** | 0 | 0 (0%) | 0 (0%) |  |
| **Stiffness on probing** | | | | |
| **Normal compared to adjacent cartilage** | 2 | 15 (71.4%) | 3 (14.3%) | 0.13 |
| **Softer** | 1 | 1 (4.8%) | 2 (9.5%) |  |
| **Very soft/hard** | 0 | 0 (0%) | 0 (0%) |  |
| **Total scores^b^** | 0-10 | 8.8 ± 0.9 | 5.2 ± 2.3 | 0.02 |

^a^Values are given as the number of the patients with percentage in parenthesis. ^b^Total scores are given as the mean ± standard deviation. Bone union group: patients who achieve bone union of the osteochondral fragment, non-union group: patients who did not achieve bone union of the osteochondral fragment.
