# Supplementary material for: Second-look arthroscopic and magnetic resonance analysis after internal fixation of osteochondral lesions of the talus
Source: Sci Rep. 2022 Jun 27;12:10833. doi: 10.1038/s41598-022-14990-5 (PMC9237059; doi:10.1038/s41598-022-14990-5)
Supplement: Supplementary file 4 — Supplementary Information 4. [file 41598_2022_14990_MOESM4_ESM.docx]

**Supplementary Table 4.** MOCART scores on MRI

| **Category** | **Points** | **No. of patients^a^** | | **p-value** |
| --- | --- | --- | --- | --- |
|  |  | **Bone union group (n=18)** | **Non-union group (n=5)** |  |
| **Volume of cartilage defect filling compared to native cartilage** | | | | |
| **Complete** | 20 | 17 (73.9%) | 2 (8.7%) | 0.01 |
| **Hypertrophy > 150 %** | 15 | 1 (4.3%) | 2 (8.7%) |  |
| **Underfilling 75-99%** | 15 | 0 (0%) | 0 (0%) |  |
| **Underfilling 50-74%** | 10 | 0 (0%) | 0 (0%) |  |
| **Underfilling 25-49%** | 5 | 0 (0%) | 1 (4.3%) |  |
| **Underfilling < 25%** | 0 | 0 (0%) | 0 (0%) |  |
| **Complete delamination** | 0 | 0 (0%) | 0 (0%) |  |
| **Integration into adjacent cartilage** | | | | |
| **Complete** | 15 | 12 (52.2%) | 0 (0%) | 0.004 |
| **Split-like defect < 2 mm** | 10 | 6 (26.1%) | 3 (13.0%) |  |
| **Defect > 2mm, but < 50%** | 5 | 0 (0%) | 0 (0%) |  |
| **Defect > 50% of length** | 0 | 0 (0%) | 2 (8.7%) |  |
| **Surface of the repair tissue** | | | | |
| **Intact** | 10 | 10 (43.5%) | 0 (0%) | <0.001 |
| **Irregularities < 50%** | 5 | 8 (34.8%) | 1 (4.3%) |  |
| **Irregularities > 50%** | 0 | 0 (0%) | 4 (17.4%) |  |
| **Structure of the repair tissue** | | | | |
| **Homogeneous** | 10 | 14 (60.9%) | 1 (4.3%) | 0.03 |
| **Heterogenous** | 0 | 4 (17.4%) | 4 (17.4%) |  |
| **Signal intensity of the repair tissue** | | | | |
| **Normal signal intensity** | 15 | 9 (39.1%) | 0 (0%) | 0.01 |
| **Minor hyperintense** | 10 | 8 (34.8%) | 2 (8.7%) |  |
| **Minor hypointense** | 10 | 1 (4.3%) | 3 (13.0%) |  |
| **Almost fluid-like** | 0 | 0 (0%) | 0 (0%) |  |
| **Close to subchondral plate** | 0 | 0 (0%) | 0 (0%) |  |
| **Bony defect or bony overgrowth** | | | | |
| **No defect or overgrowth** | 10 | 15 (65.2%) | 0 (0%) | 0.001 |
| **Defect < thickness** | 5 | 1 (4.3%) | 0 (0%) |  |
| **Overgrowth < 50%** | 5 | 1 (4.3%) | 0 (0%) |  |
| **Defect > thickness** | 0 | 0 (0%) | 2 (8.7%) |  |
| **Overgrowth > 50%** | 0 | 1 (4.3%) | 3 (13.0%) |  |
| **Subchondral changes** | | | | |
| **No subchondral changes** | 20 | 13 (56.5%) | 0 (0%) | 0.004 |
| **Edema-like < 50%** | 15 | 4 (17.4%) | 1 (4.3%) |  |
| **Edema-like > 50%** | 10 | 1 (4.3%) | 1 (4.3%) |  |
| **Cyst > 5 mm** | 0 | 0 (0%) | 2 (8.7%) |  |
| **Osteonecrosis-like signal** | 0 | 0 (0%) | 1 (4.3%) |  |
| **Total Scores^b^** | 100 | 88.3 ± 10.0 | 39.0 ± 20.4 | < 0.001 |

^a^Values are given as the number of the patients with percentage in parenthesis. ^b^Total scores are given as the mean ± standard deviation. Bone union group: patients who achieve bone union of the osteochondral fragment, non-union group: patients who did not achieve bone union of the osteochondral fragment. MOCART, Magnetic Resonance Observation of Cartilage Repair Tissue
